# A Photothermal Self-Healing Polyacrylate Gel Coating with Oxime–Carbamate Dynamic Bonds for De-Icing and Surface Protection

**DOI:** 10.3390/gels12050364

**Published:** 2026-04-27

**Authors:** Zhiwen Wang, Xuan Liu, Shuangying Wei

**Affiliations:** 1 College of Material Science and Engineering, Northeast Forestry University, Harbin 150040, China; 19163346882@163.com (Z.W.); 15545569980@163.com (X.L.); 2 Key Laboratory of Bio-Based Material Science & Technology, Northeast Forestry University, Ministry of Education, Harbin 150040, China; 3 State Key Laboratory of Woody Oil Resources Utilization, Northeast Forestry University, Harbin 150040, China

**Keywords:** gel composite coating, photothermal conversion, hydrophobic antifouling, self-healing, de-icing

## Abstract

The development of protective coatings that integrate self-healing and environmental tolerance is vital for extending substrate lifespan. In this study, a multifunctional hydrogel composite coating is developed based on a waterborne polyacrylate dynamic covalent network containing oxime–carbamate bonds. The functional monomer MEOC, which contains an oxime–carbamate dynamic bond, was synthesized and incorporated into the waterborne polyacrylate matrix to form a hydrogel network (OC-PA) with intrinsic self-healing capability. Prussian blue (PB) and nano-SiO_2_ were incorporated to form a photothermal functional layer, imparting hydrophobicity and converting light into heat for de-icing, while also activating dynamic bond rearrangement within the substrate. When the MEOC content was 7 wt% and the PB content was 2 wt%, the coating temperature rose to 110 °C within 2 min under 0.6 W/cm^2^ irradiation, and the scratch healed within 5 min. After 1 h of fracture repair, the tensile strength reached 6.68 MPa, with a repair rate as high as 92.91%, and de-icing time was reduced from 343 s to 183 s. The coating achieved a water contact angle >100°. At −20 °C, the icing delay time increased by 215%. The hydrogel coating also exhibited excellent abrasion resistance, chemical stability, UV aging resistance, and anti-fouling properties, offering a durable solution for demanding environments.

## 1. Introduction

Outdoor facilities and building materials (such as timber, metal, concrete, and plastics) frequently encounter various environmental factors affecting their surface coatings during prolonged use, including rainwater infiltration [1], ice erosion [2], mechanical damage [3], and chemical corrosion [4]. These processes not only lead to coating deterioration and loss of protective function, but may also inflict irreversible damage on the underlying substrate, thereby shortening the service life of the infrastructure [5,6]. Hence, the development of multifunctional coatings that integrate self-healing damage repair, hydrophobic anti-fouling, and active de-icing capabilities is of great significance for enhancing the environmental adaptability and durability of outdoor facilities.

In recent years, intrinsic self-healing polymers based on dynamic covalent chemistry have opened new avenues for extending coating service life [7,8,9]. In particular, oxime–carbamate dynamic bonds exhibit remarkable stability under ambient conditions while undergoing reversible dissociation at elevated temperatures to generate free isocyanate and oxime groups. This dissociation proceeds through a five-membered cyclic intramolecular transition state, resulting in low activation energy and fast reversible kinetics, making it an ideal dynamic motif for constructing self-healing polymers [10,11,12,13,14]. Waterborne polyacrylates are widely used in coating materials due to their environmental friendliness [15], excellent film-forming properties, strong adhesion to various substrates, and the tunability of glass transition temperature and mechanical properties via copolymerization [16]. Consequently, a key research challenge lies in efficiently incorporating such dynamic bonds into aqueous polyacrylate systems to impart superior self-healing ability. Such dynamic covalent networks can be regarded as covalent adaptable hydrogels when integrated into aqueous systems, offering both structural stability and reversible network rearrangement.

While self-healing functionality addresses the issue of coating integrity loss due to physical damage, outdoor coatings face a broader spectrum of environmental challenges. The accretion of ice and snow, as well as surface contamination, can severely compromise the functionality and longevity of the infrastructure [17]. Therefore, beyond damage repair, imparting additional functionalities such as water repellency and de-icing capability is crucial for comprehensive protection. In the context of water repellency and de-icing, passive hydrophobic surfaces retain an air layer via micro/nano-scale rough structures, reducing the contact area with water droplets and effectively delaying ice nucleation [17,18]. However, purely hydrophobic surfaces are prone to failure under mechanical abrasion, ice penetration, or chemical attack [19,20,21,22]. The incorporation of photothermal materials—such as carbon nanotubes [23], graphene [24], polydopamine [25], titanium nitride [26], or Prussian blue (PB) [27]—into hydrophobic coatings imparts active photothermal de-icing functionality. Among these, PB stands out as a highly promising filler owing to its strong near-infrared absorption [28], low cost [29], high photothermal conversion efficiency [30], and excellent chemical stability [31].

Although previous studies have attempted to integrate self-healing with photothermal hydrophobicity, the vast majority have relied on single-layer or homogeneous hydrogel coating systems [32]. In such designs, two fundamentally conflicting requirements are forced onto the same material. On one hand, efficient self-healing demands flexible polymer chains, low permanent crosslinking density, and reversible bond dynamics to enable network rearrangement [33]. On the other hand, durable hydrophobicity and fouling resistance require a highly crosslinked, stable micro/nanostructured surface that resists mechanical abrasion and maintains a non-wetting state [34,35]. These two sets of demands are inherently contradictory within a single-phase material. A high permanent crosslinking density, while beneficial for surface stability, severely restricts polymer segmental mobility and suppresses self-healing efficiency [36]. Conversely, optimizing dynamic bond reversibility to achieve rapid damage repair inevitably leads to frequent bond dissociation under environmental stress, compromising long-term surface integrity and hydrophobicity [37]. As a result, existing self-healing photothermal coatings have typically suffered from a mutually compromised performance, where neither function reaches its full potential. Overcoming this fundamental bottleneck to simultaneously achieve excellent self-healing and durable surface protection remains an unmet challenge.

To overcome the inherent trade-off between self-healing mobility and surface stability that plagues conventional single-layer protective coatings, we propose a spatially decoupled bilayer hydrogel architecture. Unlike prior studies that compromise either repair efficiency or mechanical robustness, our design assigns distinct, non-conflicting functions to each layer: a dynamic oxime–carbamate polyacrylate gel (OC-PA) bottom layer dedicated to efficient damage repair, and a top layer of Prussian blue/nano-SiO_2_ (SiO_2_@PB) composite that independently provides durable hydrophobicity and photothermal de-icing. This decoupling strategy allows the dynamic covalent network to retain high chain mobility for self-healing without sacrificing the surface integrity required for long-term protection. First, a functional monomer, MEOC, containing both an oxime–carbamate dynamic bond and a polymerizable terminal C=C group, was designed and synthesized. Using MEOC as a functional building block, it was successfully incorporated into an aqueous polyacrylate system via seed emulsion polymerization, yielding a hydrogel (OC-PA) with intrinsic self-healing capability. This OC-PA formulation was then employed as the self-healing substrate layer. Subsequently, a Prussian blue/nano-SiO_2_ (SiO_2_@PB) composite sol–gel was formulated and applied as the functional top layer. This spatially decoupled architecture resolves the inherent trade-off of single-layer systems by assigning distinct functions to different layers: the bottom OC-PA layer provides autonomous damage repair through dynamic bond exchange, while the functional SiO_2_@PB layer imparts durable hydrophobicity and photothermal de-icing capability via its stable micro/nano structure and efficient light-to-heat conversion. The study systematically investigates photothermal conversion, self-healing behavior, de-icing performance, and environmental tolerance of the resulting dual-layer coating, aiming to provide new design concepts and experimental evidence for the development of green, intelligent, and highly durable protective materials.

## 2. Results and Discussion

### 2.1. Thermodynamic and Mechanical Properties of OC-PA

The fundamental performance parameters of the OC-PA emulsion are summarized in Table 1. As the MEOC content increased, the average particle size of the emulsion gradually rose, yet all formulations maintained a generally excellent state of dispersion. With the exception of the sample containing 9 wt% MEOC, no precipitation was observed in the remaining emulsions after storage at room temperature for five to six months, indicating excellent storage stability.

**Figure 1:**
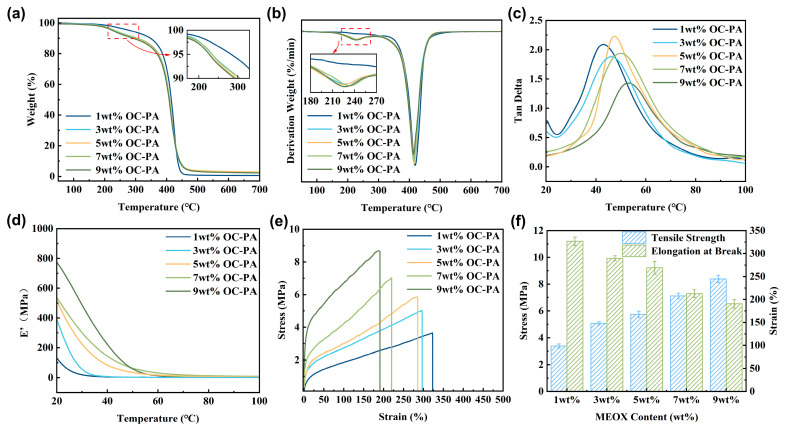
(**a**) TG and (**b**) DTG curves of OC-PA films; curves of (**c**) loss tangent (tanδ) and (**d**) storage modulus (E′) loss tangent (tanδ) with temperature of OC-PA films; (**e**) stress–strain curves and (**f**) stress–strain bar chart of different samples of OC-PA gel films.

Thermogravimetric analysis (TGA) conducted under a nitrogen atmosphere is presented in Table 2. Since the oxime–carbamate bond is a dynamic covalent bond with a bond energy typically lower than that of the acrylic ester backbone, it undergoes preferential cleavage upon heating. Consequently, the thermal decomposition temperature of the polymer system decreased slightly with increasing MEOC content [10,11,12,13,14], although the overall variation remained modest. Figure 1a,b illustrate the thermal decomposition process of OC-PA, which proceeds in two main stages. In the range of 200–400 °C, the sample exhibited approximately 20% mass loss at a relatively gentle rate, primarily attributable to the desorption of bound water and small molecules. The weight loss between 200 and 300 °C can be ascribed to the cleavage of dynamic covalent bonds. Between 350 and 450 °C, the rate of weight loss increased markedly, corresponding to the breakdown and decomposition of the polymer backbone. Dynamic mechanical analysis (DMA) curves for OC-PA are shown in Figure 1c,d. With increasing MEOC content, the glass transition temperature (Tg) of OC-PA rose from 43 °C to 53 °C, accompanied by a corresponding increase in storage modulus. This behavior is attributed to the double bonds at both ends of MEOC participating in polymerization, forming a covalent crosslinked network that restricts polymer segmental motion [36]. However, owing to the reversible cleavage and reformation of oxime–carbamate dynamic bonds, excessively high MEOC content may cause the storage modulus to decrease more rapidly with increasing temperature. Crosslinking densities of OC-PA with varying MEOC contents are listed in Table 3. Within a certain range, crosslinking density generally increased with MEOC content; however, an excessively high MEOC content led to a decrease in crosslinking density. This phenomenon can be explained by the steep rise in system viscosity caused by excessive MEOC, which severely impedes the diffusion of unreacted monomers and segments. Moreover, at high concentrations, the probability of intramolecular cyclisation reactions involving the two double bonds on the same functional monomer increases significantly. Such reactions consume double bonds without generating effective intermolecular crosslinks, resulting in wasted crosslinking sites.

The mechanical properties of OC-PA are presented in Figure 1e,f. As MEOC content increased, tensile strength gradually improved while elongation at break correspondingly decreased. At an MEOC content of 7 wt%, the OC-PA film exhibits an elongation at break of 220% and a tensile strength of 7.11 MPa. This indicates that while MEOC enhances crosslink density, it simultaneously inhibits the sliding movement of molecular chains.

These results confirm the formation of a tunable covalent gel network, where crosslinking density and segmental mobility are governed by the MEOC content. In summary, OC-PA exhibited relatively balanced comprehensive properties when the MEOC content was 7 wt%. Therefore, this composition was selected as the coating matrix gel for subsequent experiments and further investigation. Having established a self-healing-capable bottom layer, the morphology and interfacial compatibility of the composite coating system was examined next.

### 2.2. Morphological Analysis and Interface Compatibility of the Gel Coating

**Figure 2:**
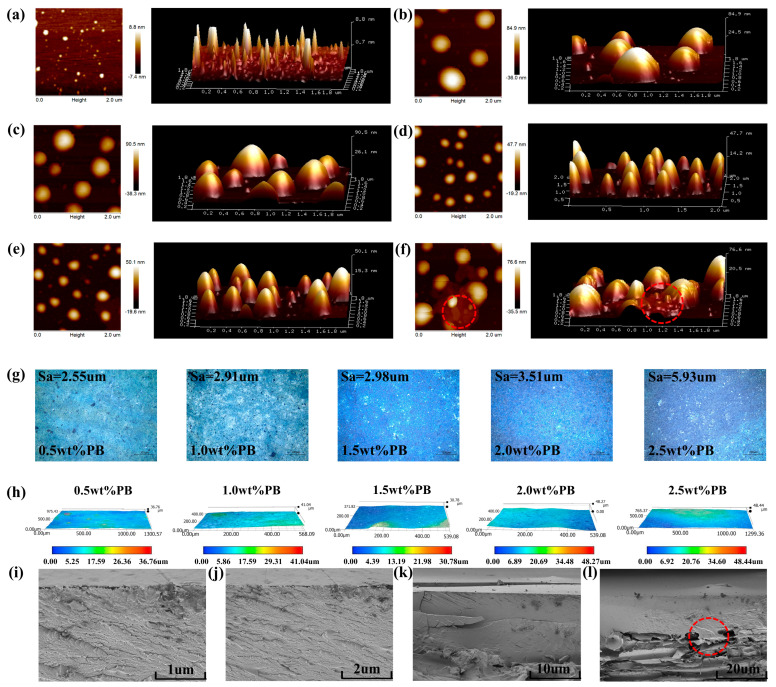
The 2D and 3D AFM phase images of (**a**) nano−SiO_2_ sol, (**b**) SiO_2_@PB_0.5_ sol, (**c**) SiO_2_@PB_1.0_ sol, (**d**) SiO_2_@PB_1.5_ sol, (**e**) SiO_2_@PB_2.0_ sol, (**f**) SiO_2_@PB_2.5_ sol; (**g**) 2D depth−of−field microscopy images of 7 wt% OC-PA/SiO_2_@PB coatings; (**h**) 3D depth−of−field microscopy images of 7 wt% OC-PA/SiO_2_@PB coatings; SEM images of the quenched fracture cross-section of ash veneer surface−coated with 7 wt% OC-PA/SiO_2_@PB_2.0_ at dimensions of (**i**) 1 μm, (**j**) 2 μm, (**k**) 10 μm, and (**l**) 20 μm.

The performance of the composite coating, especially its hydrophobicity and de-icing capability, is largely governed by surface morphology and interlayer adhesion. Accordingly, characterization was performed on the dispersion of PB in the SiO_2_ sol, surface roughness, and cross-sectional interfacial structure. Atomic force microscopy (AFM) was employed in tapping mode at room temperature to characterize the morphology of SiO_2_@PB with a focus on evaluating the dispersion of Prussian blue within the silica sol. As shown in Figure 2a–f, when the PB content was 0 wt%, 0.5 wt%, 1.0 wt%, 1.5 wt%, and 2.0 wt%, respectively, the density of the components within the sol increased and the interparticle distances decreased, but no agglomeration occurred. The sol continued to exhibit relatively uniform dispersion, whereas SiO_2_@PB_2.5_ shows markedly reduced interparticle spacing and evidence of agglomeration in certain regions. This agglomeration might be related to the addition of fumed silica, which increases the system viscosity [38]. At higher PB concentrations, particles appear to be more readily adsorbed onto the fumed silica, potentially leading to localized agglomeration and compromised dispersion uniformity.

Surface morphology and roughness of the coatings were examined using a super-depth-of-field three-dimensional microscope, with results presented in Figure 2g,h. As PB content increased, the gel coating color gradually deepened and surface roughness (Sa) increased from 2.55 μm to 5.93 μm. This is because as the PB content increases, the number of particles per unit area rises, leading to a corresponding increase in both the density and height of the protrusions, which directly results in higher surface roughness. But all values remained below 10 μm. These observations indicate the successful formation of micro–nano scale rough structures on the gel coating surface, which are conducive to achieving hydrophobicity [39].

**Figure 3:**
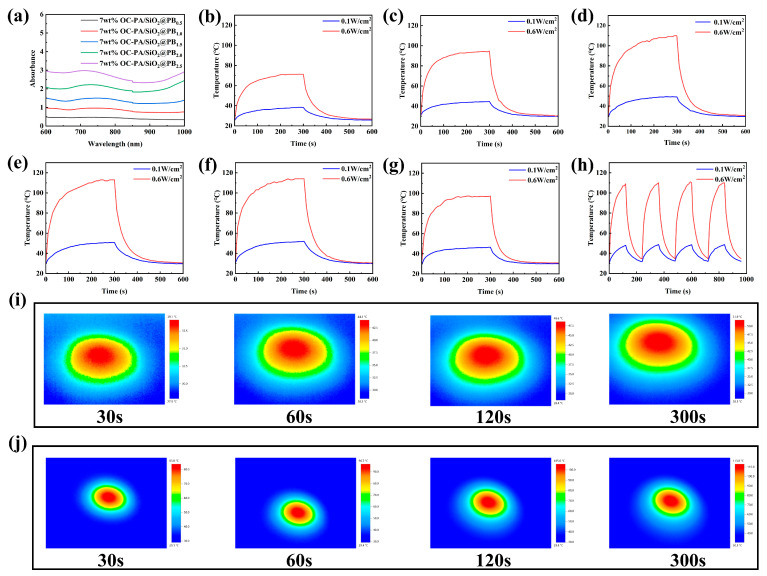
(**a**) UV-Vis absorbance map of 7 wt% OC-PA/SiO_2_@PB groups at 600–1000 nm; heating and cooling curves of (**b**) 7 wt% OC-PA, (**c**) 7 wt% OC-PA/SiO_2_@PB_0.5_, (**d**) 7 wt% OC-PA/SiO_2_@PB_1.0_, (**e**) 7 wt% OC-PA/SiO_2_@PB_1.5_, (**f**) 7 wt% OC-PA/SiO_2_@PB_2.0_, and (**g**) 7 wt% OC-PA/SiO_2_@PB_2.5_ gel films; (**h**) cyclic heating and cooling curves of 7 wt% OC-PA/SiO_2_@PB_2.0_ gel film; thermal infrared image of 7 wt% OC-PA/SiO_2_@PB_2.0_ gel film under an optical power density of (**i**) 0.1 W/cm^2^ and (**j**) 0.6 W/cm^2^.

Scanning electron microscopy (SEM) was used to examine the interfacial bonding between the gel coating and the ash veneer substrate. Figure 2i–l show the liquid nitrogen-quenched cross-sections of ash veneer coated with 7 wt% OC-PA/SiO_2_@PB_2.0_ at different magnifications and viewing angles. The images reveal a dense and uniform gel coating structure throughout, free of voids or defects, indicating that the curing process was complete and effective. The highlighted region in Figure 3j shows that the gel coating infiltrated the surface grooves of the wood, resulting in close interfacial contact and a densely packed filler morphology that is indicative of potential mechanical interlocking between the gel layer and the wood substrate. This mechanical interlocking between the gel coating and substrate [40] confers strong adhesion while simultaneously improving the surface finish of the substrate by rendering it smoother and more uniform. Notably, no visible interfacial separation was observed between the OC-PA and SiO_2_@PB layers, demonstrating excellent compatibility and interfacial bonding, which confirms the successful formation of an integrated gel composite coating.

### 2.3. The Properties and Mechanism of Photothermal Self-Healing

#### 2.3.1. Solar-Thermal Conversion

As shown in Section 2.2, the SiO_2_@PB top layer contains well-dispersed PB particles and exhibits a micro-rough surface. These features are essential for efficient photothermal conversion. Prussian blue’s high efficiency in converting light into heat stems from its unique molecular structure and electronic properties. The deep blue color corresponds to broad and intense absorption spanning the visible to near-infrared regions [41]. At the molecular level, PB forms a three-dimensional coordination network in which Fe^3+^ ions are linked to [Fe^2+^(CN)_6_]^4−^ units via cyanide bridges. Upon light absorption, an intermetallic charge transfer transition occurs from low-spin Fe^2+^ to high-spin Fe^3+^ [42]. During relaxation to the ground state, excited electrons preferentially release energy through non-radiative pathways, converting it into lattice vibrations rather than re-emitting photons. This process results in rapid and uniform heating throughout the material [43]. Moreover, the rigid three-dimensional network facilitates efficient heat generation and diffusion [44], enabling highly effective photothermal conversion.

Figure 3a shows the UV–vis absorption spectra of OC-PA/SiO_2_@PB films with varying PB contents. As the PB loading increases, absorption peaks gradually emerge and intensify in the 700–800 nm range, confirming the successful incorporation of PB into the SiO_2_ sol and its photothermal functionality within the film surface.

Real-time surface temperature monitoring was performed using a Testo 865 thermal imaging camera, and the corresponding time–temperature curves are presented in Figure 3b–g. Both the heating rate and the equilibrium temperature increased with PB content over a 5 min irradiation period. When the irradiance was 0.1 W/cm^2^, the temperature of the coating surface varied between 38.4 °C (PB content 0 wt%), 44.6 °C (PB content 0.5 wt%), 49.2 °C (PB content 1.0 wt%), 50.8 °C (PB content 1.5 wt%), 51.9 °C (PB content 2.0 wt%), and 46.5 °C (PB content 2.5 wt%). When the light intensity was 0.6 W/cm^2^, the temperature of the coating surface varied as follows: 71.3 °C (PB content 0 wt%), 94.7 °C (PB content 0.5 wt%), 110 °C (PB content 1.0 wt%), 113 °C (PB content 1.5 wt%), 114 °C (PB content 2.0%), and 97.3 °C (PB content 2.5 wt%), indicating a significant overall increase in the temperature of the coating surface. Cyclic heating tests (Figure 3h) demonstrated that the 7 wt% OC-PA/SiO_2_@PB_2.0_ gel coating reached a surface temperature of 51.9 °C after 5 min of irradiation at 0.1 W/cm^2^; under an increased power density of 0.6 W/cm^2^, the temperature rose to 114 °C within the same period, indicating excellent photo-thermal conversion stability. Thermal images of the 7 wt% OC-PA/SiO_2_@PB_2.0_ gel coating (Figure 3i,j) visually confirm its pronounced photothermal response. These results collectively demonstrate that incorporating PB into the surface layer effectively imparts superior photothermal conversion performance to the gel coating. Notably, the photothermal performance of the 7 wt%OC-PA/SiO_2_@PB_2.5_ gel coating showed a decline. One possible explanation is that excessive PB content may lead to particle agglomeration (As suggested by the AFM images in Figure 2a–f), which could enhance light scattering and thereby reduce photothermal conversion efficiency [45]. The coating’s excellent solar-thermal conversion capability is a key prerequisite for self-healing and de-icing capabilities.

#### 2.3.2. Self-Healing Properties of OC-PA/SiO_2_@PB

**Figure 4:**
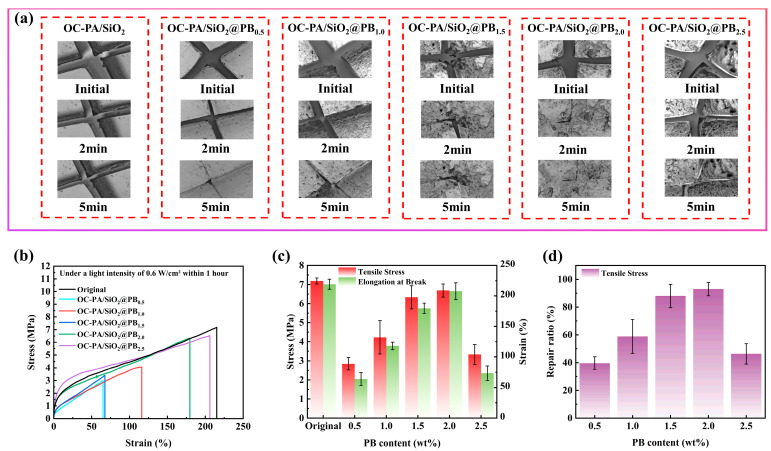
(**a**) Optical microscopy images of 7 wt% OC-PA/SiO_2_@PB gel films after scratch repair at different time intervals; (**b**) stress–strain curves, (**c**) stress–strain bar chart, (**d**) repair ratio of 7 wt% OC-PA/SiO_2_@PB gel films after fracture repair.

Optical microscopy was employed to qualitatively assess the scratch self-healing ability of OC-PA/SiO_2_@PB gel films with varying Prussian blue (PB) contents. The healing process is driven by network rearrangement of the dynamic covalent gel, facilitated by photothermal heating from the top layer. Figure 4a illustrates the evolution of pre-induced surface scratches under continuous irradiation with a xenon lamp at 0.6 W/cm^2^. After 2 min of illumination, the scratch healing efficiency progressively increased with higher PB content, attributed to enhanced photothermal conversion in the surface layer due to increased PB loading. The generated heat is transferred to the scratch interface, activating the dynamic oxime–carbamate bonds upon reaching their dissociation temperature. Under thermal stimulation, these bonds undergo reversible cleavage and recombination, triggering topological network rearrangement. Through the combined effects of surface tension and molecular segment motion, the scratch gradually closes, ultimately becoming significantly diminished or macroscopically disappearing. Notably, the self-healing efficiency of the OC-PA/SiO_2_@PB_2.5_ gel decreased under the same irradiation conditions. A plausible hypothesis is that excessive PB content may induce particle agglomeration, which could reduce photothermal conversion efficiency by increasing light scattering [45]. In addition, it is possible that aggregated PB particles might partially hinder heat transfer from the photothermal layer to the underlying polymer matrix, thereby limiting the activation of dynamic bond exchange. When the irradiation time was extended to 5 min, the scratches on all 7 wt% OC-PA/SiO_2_@PB films had essentially healed.

The film’s fracture healing capability was quantitatively evaluated via tensile test-ing. Since PB is distributed in the surface layer of the film and its content is extremely low, the mechanical properties of OC-PA/SiO_2_@PB films with different PB contents are essentially the same. Therefore, the tensile strength of the 7 wt% OC-PA/SiO_2_@PB_2.0_ film is defined as the tensile strength of the original specimen, σ_0_. The samples (30 mm × 8 mm × 1 mm) were sheared and tightly overlapped, then repaired by irradiation with a 0.6 W·cm^−2^ xenon lamp for 1 h. The results are shown in Figure 4b–d; the tensile strength of the original specimen was 7.19 MPa. As the PB content increased, the tensile strength of the film rose from 2.85 MPa to 6.68 MPa, and the repair rate increased from 39.63% to 92.99%. These results indicate that the film retains excellent mechanical properties after photothermal self-healing; 7 wt% OC-PA/SiO_2_@PB_2.5_, however, exhibits a decline in mechanical properties after healing due to reduced photothermal conversion efficiency.

Together, these results validate that the spatially decoupled design, a photothermal top layer combined with a dynamic covalent bottom layer, enables rapid, efficient healing without compromising the structural integrity of the top layer.

#### 2.3.3. Activation of Oxime–Carbamate Dynamic Bonds

**Figure 5:**
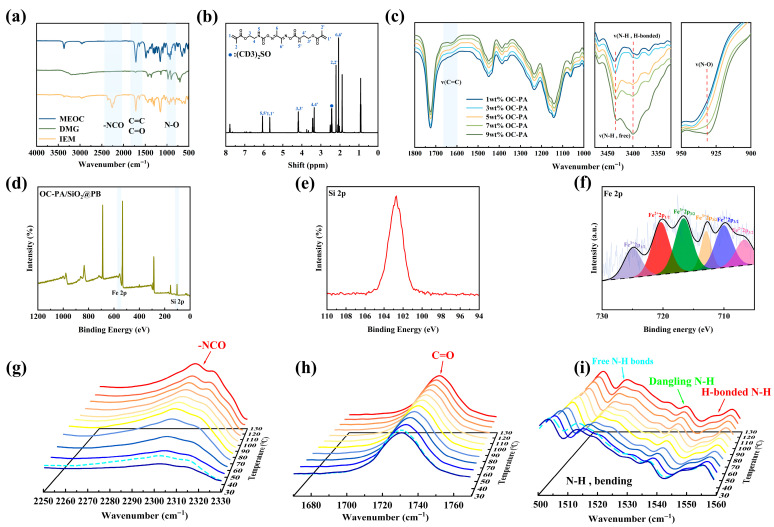
(**a**) FT-IR spectra of MEOC; (**b**) ^1^H NMR spectrum of MEOC; (**c**) FT-IR spectra of OC-PA; (**d**) XPS spectra of OC-PA/SiO_2_@PB; (**e**) XPS spectra of OC-PA/SiO_2_@PB at Si 2p; (**f**) XPS spectra of OC-PA/SiO_2_@PB at Fe 2p; in situ variable-temperature FTIR spectra of OC-PA from 30 °C to 130 °C, where are (**g**) the –NCO vibration band, (**h**) the C=O vibration band, (**i**) the N–H vibration band.

The composite coating’s excellent self-healing ability correlates well with the reversible dissociation of oxime–carbamate dynamic bonds. The oxime–carbamate dynamic bonds derive from the synthetic functional monomer MEOC. In the FT-IR spectrum of MEOC (Figure 5a), the characteristic–NCO absorption peak of IEA at 2270 cm^−1^ completely disappeared. Meanwhile, new absorption bands emerged at 3500–3300 cm^−1^, 1715 cm^−1^, 1635 cm^−1^, and 980 cm^−1^, attributed to N–H stretching, C=O stretching, C=C stretching, and N–O stretching (originating from the DMG moiety), respectively. These spectral changes collectively demonstrate the successful synthesis of MEOC. The ^1^H NMR spectrum of MEOC (Figure 5b) displays a vinyl proton signal at δ 5.69 and a methyl proton signal of the oxime group at δ 2.07, confirming the coexistence of a polymerizable carbon–carbon double bond and an oxime–carbamate dynamic bond within the molecule. For the OC-PA series (Figure 5c), a broad and intense absorption band at 1630 cm^−1^, corresponding to the stretching vibration of C=C unsaturated double bonds, progressively intensified with increasing MEOC content. Absorption peaks at 3435 cm^−1^ and 3400 cm^−1^ are assignable to free N–H and hydrogen-bonded N–H stretching vibrations, respectively; both also increased with MEOC loading, reflecting the presence of both unsaturated C=N and saturated N–H structures in the incorporated MEOC units. Additionally, the band at 930 cm^−1^, attributed to N–O stretching, grew concomitantly. The systematic increase in intensity of all these characteristic bands with MEOC content confirms the successful incorporation of MEOC into the polyacrylate backbone.

Figure 5d shows the broad XPS scan spectrum of the functional layer. Figure 5e shows the high-resolution XPS scanning spectrum of the Si 2p peak, where the peak center of Si 2p is approximately 102.8 eV, indicating that it corresponds to Si^4+^ [46]; Figure 5f shows the high-resolution XPS scan spectrum of the Fe 2p peak. Due to the complexity of the valence states of Fe, the Fe 2p peak exhibits an irregular shape, but its general range is 700–730 eV, indicating the presence of Fe^2+^ and Fe^3+^ [47]. These results confirm the presence of silicon dioxide and PB in the functional layer.

Under a specific light intensity, the PB in the functional layer converts light energy into heat, which is then transferred to the OC-PA substrate layer. This leads to rapid heating of the substrate over a short period, thereby activating the oxime–carbamate dynamic bonds. To elucidate the self-healing mechanism, the reversible behavior of the OC-PA dynamic network was investigated using in situ temperature-dependent infrared spectroscopy, using the sample with 7 wt% MEOC as a representative example. As shown in Figure 5g, both the oxime–carbamate dynamic bonds and hydrogen bonds within OC-PA undergo reversible dissociation upon heating [10,48]. When the temperature increased from 30 °C to 130 °C, characteristic absorption peaks corresponding to NCO gradually appeared in the 2330–2290 cm^−1^ range, indicating thermally induced cleavage of the oxime–carbamate bonds. Figure 5h presents the infrared response of the C=O bond. As the temperature rises, hydrogen bond dissociation releases previously constrained C=O groups, enhancing their vibrational freedom and resulting in intensified absorption peaks that shift toward higher wavenumbers (blue shift). Figure 5i depicts the evolution of N–H bending vibrations. The intensities of the bands corresponding to hydrogen-bonded N–H (1560–1545 cm^−1^) and free N–H (1520–1510 cm^−1^) both decrease with increasing temperature. This behavior arises because some N–H bonds adopt a “dangling state” intermediate between bound and free forms during polymer chain conformational restriction and dynamic rearrangement [49]. The absorption peak observed in the 1540–1535 cm^−1^ range, which intensifies with rising temperature, provides evidence for the existence of this dangling state. Upon subsequent cooling back to 30 °C, the characteristic peaks of NCO, C=O, and N–H gradually recover to their original intensities, conclusively demonstrating the excellent thermal reversibility of the dynamic network. The reversible dissociation and recombination of oxime–carbamate bonds under thermal stimulation are the molecular basis for the covalent adaptable gel behavior of the OC-PA network.

### 2.4. De-Icing Performance of OC-PA/SiO_2_@PB

**Figure 6:**
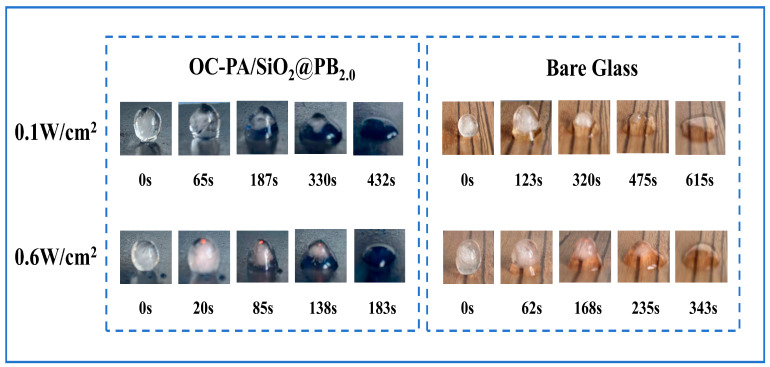
Ice sphere melting process of OC-PA/SiO_2_@PB_2.0_ gel coating at different light intensities.

The OC-PA/SiO_2_@PB gel coating achieves passive anti-icing through the hydrophobicity imparted by surface SiO_2_, while also enabling active de-icing via the photothermal conversion properties of PB. Building on the previously demonstrated photothermal performance, the de-icing capability of the gel coating was further evaluated. As shown in Figure 6, under a light power density of 0.1 W/cm^2^, the control group required 615 s for complete ice melting, whereas the OC-PA/SiO_2_@PB_2.0_ gel coating achieved complete melting in 432 s, which represents a reduction of approximately one-third. When the light power density was increased to 0.6 W/cm^2^, the melting time for the coated sample further decreased to 183 s, a reduction of about half compared to the control. These results indicate that the photothermal conversion efficiency of PB improves with increasing light intensity, and the active de-icing performance of the coating is correspondingly enhanced.

### 2.5. Surface Protection and Hydrophobic Anti-Soiling Performance

**Figure 7:**
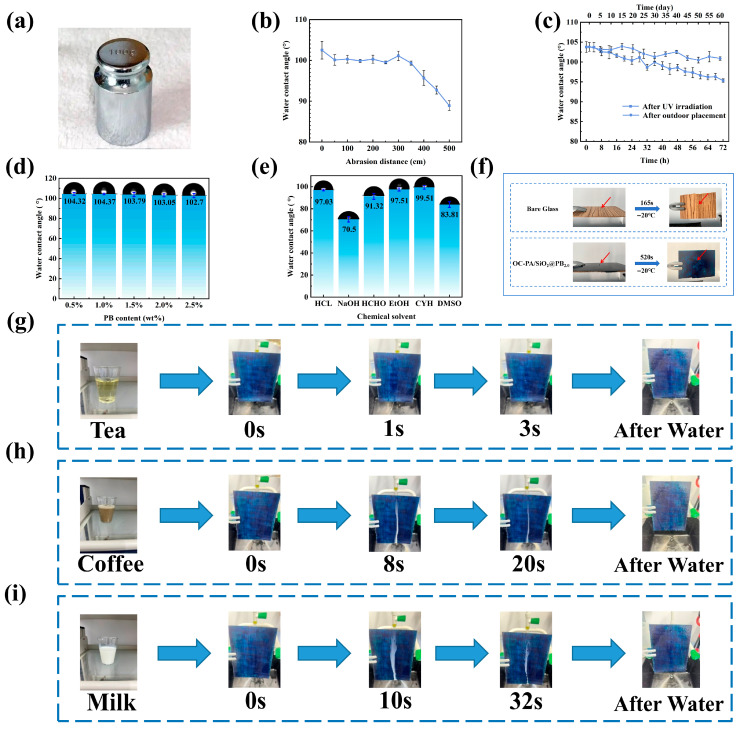
(**a**) Physical image of a 100 g weight; (**b**) changes in WCA after several rounds of sandpaper abrasion with a 100 g applied load; (**c**) changes in WCA after UV irradiation and outdoor placement; (**d**) changes in WCA after immersion in various chemical solvents; (**e**) water contact angle of OC-PA/SiO_2_@PB; (**f**) droplet freezing process; (**g**) tea, (**h**) coffee, and (**i**) milk were poured onto the surface of the OC-PA/SiO2@PB gel coatings.

A practical protective coating must retain its functionality even when exposed to external environmental conditions. The adhesion and hardness of gel coatings with varying Prussian blue (PB) contents are summarized in Table 4. All gel coatings exhibited excellent adhesion and hardness, establishing a foundation for their outstanding mechanical stability. Figure 7a shows the abrasion resistance test setup, with results presented in Figure 7b. After 100 abrasion cycles (cumulative friction distance of 500 cm), the water contact angle (WCA) of the gel coating remained stable at approximately 90°, indicating that hydrophobicity was maintained and suitable for practical use. Figure 7c illustrates the weathering resistance under two illumination conditions: after 72 h of UV irradiation at 15 mW/cm^2^, the WCA decreased by about 8°; after 60 days of exposure to natural mixed light, the WCA dropped by less than 3°. In both cases, the WCA remained above 90°, demonstrating excellent light aging resistance and suitability for long-term outdoor applications.

As shown in Figure 7d, the WCA values of OC-PA/SiO_2_@PB gel coatings with different PB contents all exceeded 100°, confirming favorable hydrophobicity [50]. Chemical stability was evaluated by immersing the gel coatings in various reagents (Figure 7e). After immersion in HCl solution (pH = 1), the gel coating retained a WCA of 97°, indicating strong acid resistance. However, the hydrophobicity of the coating has decreased significantly in a sodium hydroxide solution (pH = 14), with a water contact angle of only 70.5°. This is attributed to the dissolution of surface SiO_2_ into silicates under alkaline conditions, combined with the hydrolysis of the underlying oxime–carbamate dynamic bonds [51], leading to crosslinked network failure and accelerated degradation. The gel coating maintained hydrophobicity in most organic solvents, though exposure to weakly alkaline dimethyl sulfoxide reduced the WCA below 90°.

Hydrophobic surfaces with high water contact angles cause water droplets to contract into spherical shapes, minimizing solid–liquid contact area. Since ice nuclei preferentially form at this interface, a reduced contact area lowers nucleation site density and raises the energy barrier for ice nucleation, effectively delaying icing [17,18]. Using the 7 wt% OC-PA/SiO_2_@PB_2.0_ gel coating as a representative sample, its anti-icing performance was compared with that of uncoated ash veneer (Figure 7f). Water droplets froze completely within 165 s on the bare surface, whereas freezing took 520 s on the coated surface, an improvement of approximately 215%. This demonstrates the gel coating’s outstanding passive anti-icing capability.

The anti-fouling performance was further assessed using common liquid contaminants, including tea, coffee, and milk (Figure 7g–i). After tea spillage, the gel coating surface remained clean; coffee and milk left only faint traces, both of which were completely removed by rinsing with water. These results confirm that the OC-PA/SiO_2_@PB gel coating possesses excellent surface protection properties.

## 3. Conclusions

This study presents a dual-layer photothermal waterborne gel coating designed using a spatial decoupling strategy to integrate hydrophobic antifouling, self-healing, and active de-icing functions. The bottom layer comprises a polyacrylate network with dynamic oxime–carbamate bonds (OC-PA), while the top layer consists of a Prussian blue/nano-SiO_2_ (SiO_2_@PB) composite that provides photothermal conversion and hydrophobicity. This architecture resolves the inherent trade-off between self-healing mobility and surface stability in single-layer systems.

An optimal MEOC content (7 wt%) in the OC-PA matrix yielded balanced thermomechanical properties (crosslinking density: 1845.61 mol·m^−3^, Tg: 50.08 °C, tensile strength: 7.10 MPa). In situ FTIR confirmed the reversible dissociation of oxime–carbamate bonds under thermal stimulation. The SiO_2_@PB layer imparted hydrophobicity (WCA > 100°), delaying freezing to 520 s at −20 °C. Under 0.6 W/cm^2^ irradiation, the gel coating reached 110 °C within 2 min, and the scratch healed in 5 min. After 1 h of fracture repair, the tensile strength reached 6.68 MPa, with a repair rate as high as 92.91%, and de-icing time was reduced from 343 s to 183 s. The gel coating also exhibited excellent adhesion (grade 0–1), hardness (3–4H), abrasion resistance, chemical stability, and antifouling properties.

This work validates an effective spatial decoupling strategy for developing durable, multifunctional protective gel coatings suitable for demanding outdoor applications.

## 4. Materials and Methods

### 4.1. Materials

2-Isocyanatoethyl methacrylate (IEM), methyl methacrylate (MMA), butyl acrylate (BA), acetoacetoxyethyl methacrylate (AAEM), sodium dodecyl sulfate (SDS), ammonium persulfate (APS), methyl ethyl ketone (MEK), Prussian blue (PB), and fumed nano-silica were purchased from Shanghai Macklin Biochemical Technology Co., Ltd. (Shanghai, China). OP-10 emulsifier, sodium hydroxide (NaOH), dimethylglyoxime (DMG), and dibutyltin dilaurate (DBTDL) were obtained from Aladdin Reagent Co., Ltd. (Shanghai, China). Nano-silica sol was supplied by Shanghai Huaxia Jiahe New Material Technology Co., Ltd. (Shanghai, China). Deionized water was prepared in the laboratory.

### 4.2. Methods

#### 4.2.1. Synthesis of Functional Monomer MEOC

**Scheme 1:**
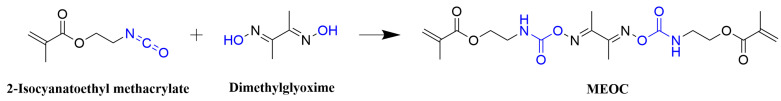
Synthesis route of MEOC.

Dimethylglyoxime (5.806 g, 50 mmol), dibutyltin dilaurate (0.04 g), and 4-methoxyphenol (0.4 g) were dissolved in acetone (160 mL) and stirred at 45 °C until complete dissolution to obtain Solution A. 2-Isocyanatoethyl methacrylate (15.515 g, 10 mmol) was dissolved in acetone (40 mL) to prepare Solution B. Under a nitrogen atmosphere, Solution B was added dropwise to Solution A using a constant-pressure dropping funnel from Aladdin Reagent Co., Ltd. (Shanghai, China). After reacting at 45 °C for 3 h, an additional portion of DBTDL (0.04 g) was introduced, and the reaction mixture was stirred for a further 2.5 h. Upon completion, the reaction mixture was collected, and the solvent (acetone) was removed by rotary evaporation under reduced pressure. The obtained product, MEOC, was stored in a refrigerator at 4 °C until further use. The reaction mechanism is shown in Scheme 1.

#### 4.2.2. Synthesis of OC-PA Materials Containing Oxime–Carbamate Dynamic Bonds

Methyl methacrylate (MMA) and butyl acrylate (BA) were washed repeatedly with a 3 wt% NaOH solution to remove the polymerization inhibitor. The monomers were then rinsed several times with deionized water to eliminate any residual alkaline solution, followed by drying over anhydrous sodium sulfate for 24 h to obtain purified MMA and BA.

Separate mixtures were prepared by weighing specific amounts of monomers. For the seed layer, MMA (3.60 g) and BA (2.40 g) were combined and mixed thoroughly. For the shell layer, MMA (20.40 g), BA (17.60 g), AAEM (6.00 g), and MEOC (at loadings of 1 wt%, 3 wt%, 5 wt%, 7 wt%, or 9 wt% relative to total monomer mass) were weighed and homogenized.

**Scheme 2:**
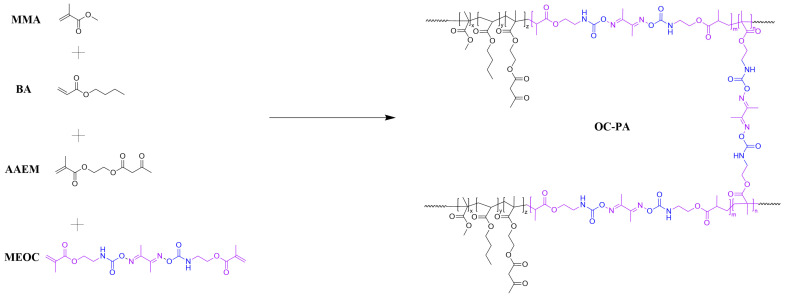
Synthesis route of OC-PA.

An emulsifier solution was prepared by dissolving OP-10 (0.60 g) and SDS (1.20 g) in deionized water (54.0 g). The solution was transferred to a four-neck flask purged with nitrogen and stirred at 350 rpm in an 80 °C water bath for 1 h. An initiator solution was prepared by dissolving APS (0.24 g) in deionized water (15.0 g). The pre-weighed shell monomer mixture, along with 10 wt% of the initiator solution, was added to the flask. The mixture was stirred for 15 min, yielding a pale blue seed emulsion. Subsequently, the remaining shell monomer mixture and initiator solution were added dropwise to the flask using a peristaltic pump, at rates of one drop every 6 s and one drop every 50 s, respectively. After the additions were complete, the reaction mixture was cooled to 75 °C and stirred for an additional 3 h to obtain the OC-PA emulsion. The obtained OC-PA emulsion can be cast into free-standing gel films upon water evaporation, forming a crosslinked polyacrylate gel network containing dynamic oxime–carbamate bonds. The reaction mechanism is shown in Scheme 2.

To prepare gel films for characterization, a predetermined amount of the OC-PA emulsion was cast into a polytetrafluoroethylene mold. After standing at room temperature for 24 h, the mold was placed in a forced-air oven at 30 °C for another 12 h to remove residual water, yielding solid OC-PA gel films.

#### 4.2.3. Preparation of SiO_2_@PB Composite Sol

A specified amount of Prussian blue (PB) was subjected to ball milling at 30 kW for 5 min to obtain PB particles with a size range of approximately 200–300 nm. The resulting PB particles (0.075 g, 0.150 g, 0.225 g, 0.300 g, and 0.375 g, corresponding to 0.5 wt%, 1.0 wt%, 1.5 wt%, 2.0 wt%, and 2.5 wt% relative to the final composite, respectively) were weighed and separately added to 10 mL of ethanol. Each suspension was then ultrasonically dispersed at 300 W for 30 min to obtain homogeneous PB suspensions. Subsequently, 5 g of nano-silica sol was introduced into each suspension and stirred magnetically at 300 rpm for 30 min at room temperature. Finally, 0.1 g of fumed silica was slowly added, and the mixture was stirred magnetically at 600 rpm for an additional 15 min to yield the SiO_2_@PB composite sol.

#### 4.2.4. Preparation of OC-PA/SiO_2_@PB Gel Coatings

**Figure 8:**
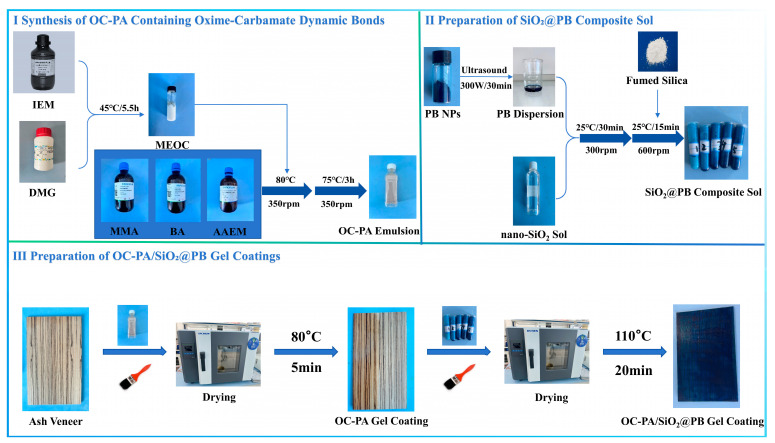
Preparation process of the OC-PA/SiO_2_@PB gel coating films.

The OC-PA emulsion (96 wt% of the total formulation) was used as the matrix resin. To this, emulsifier (1.5 wt%), aqueous defoamer (0.5 wt%), substrate wetting agent (0.8 wt%), thickener (0.7 wt%), and water-based leveling agent (0.5 wt%) were sequentially added. The mixture was stirred at 1200 rpm for 30 min to obtain a self-healing wood coating formulation. The resulting gel coating was uniformly applied onto the surface of sanded ash veneer. The coated veneer was then dried in an oven at 80 °C for 5 min to remove moisture and form the first layer. This coating and drying process was repeated three times to build up a uniform gel film. The resulting OC-PA layer functions as a dynamic covalent gel substrate. Subsequently, the prepared SiO_2_@PB composite sol was brush-applied onto the surface of the OC-PA coated veneer and dried in an oven at 110 °C for 10 min, ultimately yielding the OC-PA/SiO_2_@PB gel composite coating. The preparation process is shown in Figure 8.

### 4.3. Characterization and Testing

#### 4.3.1. Structural Analysis

The chemical structures of the synthesized MEOC monomer and the polyacrylate samples were characterized by Fourier transform infrared spectroscopy (FTIR) using a Nicolet iS50 instrument over a wavenumber range of 4000–500 cm^−1^ at a resolution of 2 cm^−1^.

^1^H NMR spectra of MEOC were recorded on a Bruker 400 MHz nuclear magnetic resonance spectrometer in deuterated dimethyl sulfoxide (DMSO-*d*_6_).

The surface of the OC-PA/SiO_2_@PB gel film was analyzed using a Thermo Scientific K-Alpha X-ray photoelectron spectrometer.

The dynamic reversibility of oxime–carbamate bonds and hydrogen bonds in the OC-PA gel film was investigated by in situ infrared analysis (VERTEX 80v) in ATR mode. Spectra were recorded at 10 °C intervals from 30 °C to 130 °C and then back to 30 °C, with heating and cooling rates of 5 °C/min.

#### 4.3.2. Emulsion Performance Testing

The OC-PA emulsion was diluted to 1 wt% with deionized water and ultrasonicated for 10 min. The supernatant was then analyzed for particle size distribution at room temperature using a Zetasizer Nano Series (Malvern Instruments, Shanghai, China). Emulsion stability was assessed by visually monitoring any changes in appearance and measuring the time over which the emulsion remained stable in a transparent container.

#### 4.3.3. Thermodynamic Performance Testing

Thermal stability of OC-PA films was evaluated by thermogravimetric analysis (TG, TG209F3 Nevio, NETZSCH-Gerätebau GmbH, Selb, Germany) under a nitrogen atmosphere from 30 to 800 °C at a heating rate of 20 °C/min.

The glass transition temperature (Tg) and modulus evolution were characterized by dynamic mechanical thermal analysis (TA-Q800, TA Instruments, New Castle, DE, USA) in tensile mode at a frequency of 1 Hz, a heating rate of 10 °C/min, over a temperature range of 0–180 °C.

Crosslink density (νₑ) was calculated using the equation:

(1) $$Ve=\frac{E’}{3RT}$$

where νₑ is the crosslink density (mol·m^−3^), E′ is the storage modulus (Pa) at temperature T (K), T is taken as Tg + 20 °C, and R = 8.314 J·mol^−1^·K^−1^.

#### 4.3.4. Mechanical Property Testing

Tensile strength and elongation at break of the gel films were measured at room temperature using a universal testing machine (TH-8203S, Tuobo Machinery Equipment Co., Ltd, Suzhou, China) at a strain rate of 10 mm/min. Each sample was tested three times in parallel.

#### 4.3.5. Photothermal Conversion Testing

Transmittance of the 7 wt% OC-PA/SiO_2_ and 7 wt% OC-PA/SiO_2_@PB gel films (thickness: 1 mm) was measured using a UV–visible spectrophotometer (TU-1950, Beijing Puxi General Instrument Co., Ltd, Beijing China) over a wavelength range of 190–1100 nm with a 1 nm scanning interval.

Coatings with varying PB contents were irradiated with a xenon lamp(BBZM-III, Anhui Bobei Lighting Electrical Appliance Factory, Xuancheng, China) at two power densities (0.1 W/cm^2^ and 0.6 W/cm^2^). Real-time temperature evolution and thermal images were recorded using a Testo 865 thermal imaging camera (German Instruments International Trading (Shanghai) Co., Ltd, Shanghai, China).

#### 4.3.6. Self-Healing Performance Testing

Surface scratch healing tests were performed to assess self-healing behavior. A through-scratch (width ≈ 80 μm) was created on the surface of a 1 mm thick gel film using a blade. OC-PA/SiO_2_@PB gel films with different PB contents were then heated under a xenon lamp, and morphological changes in the scratched region were observed and documented by optical microscopy.

The self-healing properties of OC-PA/SiO_2_@PB gel films were investigated using a TH-8203S electronic universal testing machine. Rectangular specimens (30 mm × 8 mm × 1 mm) were cut in half along the center using a safety knife. The two halves were then immediately pressed together under a certain pressure and exposed to a xenon lamp (0.6 W) for 1 h to facilitate self-healing. Each sample was tested three times in parallel. The fracture healing rate (η), used to quantify the healing effect, was defined as the ratio of the tensile strength of the healed specimen (σt) to the tensile strength of the original specimen (σ0):

(2) $$\eta =\frac{\sigma_{t}}{\sigma_{0}}\times 100\%$$

#### 4.3.7. De-Icing Performance Testing

Ice-melting capability was evaluated by a photothermal ice ball melting test. Ice balls (diameter 1 cm) prepared in a circular mould were placed on the 7 wt% OC-PA/SiO_2_@PB gel coating surface and irradiated with simulated sunlight from a xenon lamp at power densities of 0.1 W/cm^2^ and 0.6 W/cm^2^. The melting process was continuously recorded until complete liquefaction. Uncoated ash veneer was used as a control.

#### 4.3.8. Microstructural Characterisation

The morphology of the SiO_2_@PB sol was examined by atomic force microscopy (AFM, Bruker Multimode 8, Bruker Corporation, Billerica, MA, USA) in tapping mode at room temperature. To evaluate the dispersion and agglomeration of Prussian blue within the composite sol, samples with different PB contents were diluted with deionized water at equal ratios, drop-cast onto 1 cm × 1 cm silicon substrates, and dried at 50 °C for 30 min prior to imaging.

Interfacial adhesion between the 7 wt% OC-PA/SiO_2_@PB gel coating and the wood substrate, as well as the internal interfacial bonding within the composite gel coating, was characterized by scanning electron microscopy (SEM, GeminiSEM 300, Carl Zeiss AG, Oberkochen, Germany) with a scale bar of 2 μm for all images.

Surface topography and roughness of the 7 wt% OC-PA/SiO_2_@PB gel coating were examined using a super-depth-of-field three-dimensional microscope system (image scale: 200 μm).

#### 4.3.9. Surface Protection and Hydrophobic Anti-Soiling Performance Testing

Paint film adhesion was evaluated by the cross-cut method according to GB/T 9286–2021 [52], with ratings from 0 (best) to 5 (worst). Three parallel tests were conducted at different locations on each sample, and the results were averaged.

GB/T 6739–2006 [53]. A pencil of selected hardness was mounted in the fixture and moved horizontally at constant speed along the slide rail to scratch the film surface. The hardness grade was assessed based on the presence of scratches. Each sample group was tested three times, and the arithmetic mean was taken.

Abrasion resistance was tested using the sandpaper friction method. A 7 wt% OC-PA/SiO_2_@PB_2.0_-coated ash veneer was abraded with 600-grit sandpaper under a 100 g load; each friction pass covered approximately 5 cm. One cycle corresponded to a cumulative friction distance of 50 cm, and a total of 10 cycles were performed. Fresh sandpaper was used for each cycle, and the water contact angle (WCA) was measured after each cycle.

UV ageing resistance was evaluated by exposing the ash veneer coated with the 7 wt% OC-PA/SiO_2_@PB_2.0_ formulation to ultraviolet irradiation. A UV lamp was positioned vertically 2 cm above the gel coating surface, delivering a power density of approximately 15 mW/cm^2^. WCA was recorded every 4 h over 72 h. To simulate outdoor ageing, samples were exposed to natural light for 60 days (temperature range −10 to −27 °C, UV index 25), with WCA measurements taken every 5 days. Each sample was tested three times in parallel.

Wetting behavior was characterized by the sessile drop method using a JC2000 contact angle instrument (Shanghai Zhongchen Technology Equipment Co., Ltd, Shanghai, China). Droplets were deposited onto three distinct regions of each specimen; contour images were captured and contact angles calculated. Each sample was tested three times in parallel.

Chemical resistance was evaluated by fully immersing the ash veneer coated with the 7 wt% OC-PA/SiO_2_@PB_2.0_ in various media: hydrochloric acid (pH = 1), sodium hydroxide (pH = 14), formaldehyde solution, acetic acid solution, ethanol solution, cyclohexane solution, and dimethyl sulfoxide solution. Coating stability was assessed by monitoring changes in WCA. Each sample was tested three times in parallel.

Anti-icing performance was investigated using the water droplet freezing method. At −20 °C in an outdoor environment, a 10 µL droplet of deionized water was placed on the coated surface, and the time required for complete freezing was recorded. Uncoated ash veneer served as the control.

Anti-fouling performance was assessed by pouring common liquid contaminants (tea, milk, and coffee) sequentially onto the 7 wt% OC-PA/SiO_2_@PB_2.0_-coated surface and observing any residual contamination.

## Figures and Tables

**Table 1:** Emulsion properties of OC-PA.

Sample Codes	Solid Content (wt%)	Average Particle Size (nm)	Zeta Potential (mV)	PDI	Appearance	Storage Stability (Months)
1 wt% OC-PA	38.75	146.4	−47.4	0.096	Milky white	>6
3 wt% OC-PA	40.09	193.2	−43.1	0.117	Orange	>6
5 wt% OC-PA	40.43	278.6	−41.7	0.194	Pale pink	>6
7 wt% OC-PA	41.11	354.7	−41.5	0.243	Beige	>5
9 wt% OC-PA	41.64	564.2	−40.7	0.322	Pink	>2

**Table 2:** Characteristic thermal degradation temperatures of OC-PA gel films.

Sample Codes	T_d5%_/°C	T_d10%_/°C	T_d50%_/°C	T_max_/°C
1 wt% OC-PA	283.235	348.235	415.235	418.801
3 wt% OC-PA	234.648	306.479	409.524	413.674
5 wt% OC-PA	233.504	306.313	409.498	415.376
7 wt% OC-PA	228.924	298.22	409.573	414.489
9 wt% OC-PA	225.738	292.119	407.53	413.432

**Table 3:** Glass transition temperature, storage modulus, and crosslinking density of OC-PA gel films with different MEOC contents.

Sample Codes	Tg/°C	E′ (25 °C)/MPa	E′ (Tg + 20 °C)/MPa	Ve/mol·m^−3^
1 wt% OC-PA	42.87	49.83	1.39	165.85
3 wt% OC-PA	45.99	177.82	1.94	229.35
5 wt% OC-PA	47.41	347.81	11.16	1313.83
7 wt% OC-PA	50.08	410.88	15.80	1845.61
9 wt% OC-PA	53.04	639.96	5.71	661.29

**Table 4:** Adhesion and Hardness of the OC-PA/SiO_2_@PB gel Coatings.

Sample	Adhesion	Hardness
7 wt% OC-PA/SiO_2_@PB_0.5_	Level 0	3H
7 wt% OC-PA/SiO_2_@PB_1.0_	Level 1	3H
7 wt% OC-PA/SiO_2_@PB_1.5_	Level 1	4H
7 wt% OC-PA/SiO_2_@PB_2.0_	Level 1	4H
7 wt% OC-PA/SiO_2_@PB_2.5_	Level 1	4H

## Data Availability

The original contributions presented in this study are included in the article. Further inquiries can be directed to the corresponding author.
